# Functional Evaluation of Computationally Designed IL-10 in IL-10 KO Mice

**DOI:** 10.3390/biom16030482

**Published:** 2026-03-23

**Authors:** Jordan Stokes, Iram Hyder, Zhihang Shen, Peter Ramdhan, Allison Bayer, Clive Wasserfall, Chenglong Li, Sihong Song

**Affiliations:** 1 Departments of Pharmaceutics, University of Florida College of Pharmacy, Gainesville, FL 32610, USA; jordan.stokes@ufl.edu (J.S.); ihyder@ufl.edu (I.H.); 2 Department of Medicinal Chemistry, University of Florida College of Pharmacy, Gainesville, FL 32610, USA; zhihangshen@ufl.edu (Z.S.); pramdhan1@ufl.edu (P.R.); 3 Department of Pathology, Immunology and Laboratory Medicine, University of Florida College of Medicine, Gainesville, FL 32610, USA; allisonbayer@ufl.edu (A.B.); wasserfa@pathology.ufl.edu (C.W.)

**Keywords:** inflammatory bowel disease, recombinant adeno-associated virus vector, Interleukin 10 (IL-10), computational design of a protein drug and gene therapy

## Abstract

Studies have shown that IL-10 has therapeutic potential for inflammatory diseases. However, it is challenging to use IL-10 as a therapeutic drug because it also possesses pro-inflammatory functions. To reduce these pro-inflammatory effects of IL-10, we have designed three IL-10 mutants using structure-based computational design technology. We demonstrated that these mutants exhibited significantly lower activity in IL-10-responsive cell lines than wild-type IL-10. Using recombinant adeno-associated virus (rAAV8) vectors expressing wild-type or mutant IL-10 molecules, we performed gene therapy experiments in IL-10 KO mice. The results showed that our vectors mediated high levels of transgene expression. Importantly, IL-10 gene therapy increased body weight gain, reduced colon injury, and prevented the development of inflammatory bowel disease (IBD). Moreover, IL-10 mutant gene therapy elicited significantly lower stimulation of CD8 T and NK cells compared with the wild-type IL-10 group. In summary, our IL-10 mutants provide a protective effect comparable to wild-type IL-10 in the IL-10 KO mouse model, suggesting that they may potentially have reduced pro-inflammatory function. While rigorous investigations of safety and efficacy in different disease models will be required, these results indicate the therapeutic potential of IL-10 mutant gene therapy for inflammatory diseases such as IBD.

## 1. Introduction

Inflammatory bowel disease (IBD) is an autoimmune disease that causes chronic scarring and inflammation in the gastrointestinal tract. IBD comprises two main subtypes: Crohn’s disease and ulcerative colitis. Crohn’s disease results in patchy inflammation that can spread throughout both the small and large intestines, whereas ulcerative colitis is confined to the large intestine. Patients with IBD often experience persistent diarrhea, abdominal pain, rectal bleeding or bloody stools, weight loss, and fatigue. More than 3.1 million people are diagnosed with IBD [1]. Current therapies help maintain remission and ameliorate the disease’s secondary effects, but they do not cure IBD [2,3]. Because the available drugs have limited efficacy and can cause numerous side effects, developing more effective and safer treatments is imperative [4].

The etiology of IBD is complex and multifactorial, including dietary, environmental, microbial, immunological, and genetic factors. It has been shown that IL-10, IL-1R2, CARD9, NOD2, and IL-23 play important roles in the pathogenesis of IBD [5,6]. Genetic studies have shown that the IL-10 signaling pathway is highly associated with IBD development. Patients with defects in the IL-10 signaling pathway develop life-threatening colitis. Although IL-10 is generally considered as an anti-inflammatory and immunoregulatory cytokine, its therapeutic application is limited by its pleiotropic nature, the pro-inflammatory effect of stimulating effector T cells to produce interferon-γ(IFN-γ) and granzyme B [7,8]. A recent study determined the structure of the complete IL-10 and its receptor complex (the hexameric IL-10/IL-10Rα/IL-10Rβ complex), providing a blueprint for the design of IL-10 mutants [9]. Importantly, this study provides insight into generating potentially therapeutic IL-10 mutant(s), which retain normal anti-inflammatory effects but have no or lower pro-inflammatory effects.

In this study, we used recently developed computational design technology for selecting IL-10 mutants, employed recombinant adeno-associated virus vector (rAAV) for delivery, and tested therapeutic effects in an IL-10 knockout (KO) mouse model [10,11,12]. We showed that selected IL-10 mutants exhibited reduced activity in vitro compared with wild-type IL-10. In the IL-10 KO mouse model, rAAV8-mediated delivery of human IL-10 mutants fully corrected the defects associated with IBD. These results suggest that the IL-10 mutants and their expressing rAAV vectors hold promise for future studies in developing anti-inflammatory therapy.

## 2. Materials and Methods

### 2.1. Structure Preparation

The initial structure of interleukin-10 (IL-10) was obtained from the Protein Data Bank (PDB ID: 6X93) [9], which contains IL-10 bound to the IL-10 receptor α and β subunits in a hexameric assembly. To reduce computational cost, one trimeric unit was removed, yielding a single trimeric complex for all subsequent simulations. Protein preparation was carried out using Schrödinger Maestro’s Protein Preparation Wizard [13], including the addition of missing atoms, assignment of bond orders, optimization of hydrogen-bonding networks, and assignment of protonation states at pH 7.4 ± 2.0. Missing residues were rebuilt during this process. Mutant models were generated by manually introducing the R32D and E96R point mutations individually and in combination. For the models with a human ligand bound to a mouse receptor, the mouse receptor was constructed using homology modeling via the SWISS-MODEL server [14].

### 2.2. Simulation Parameters

Molecular dynamics (MD) simulations were performed using the GPU implementation of pmemd.cuda [15,16] from AMBER20 [17] using the ff19SB [18] parameters for the protein. The systems were solvated in a truncated octahedron using the OPC [19] water model with a 15.0 Å buffer. Sodium and chloride ions were added to neutralize the system and mimic a physiological salt concentration of 150 mM NaCl.

Each system was subjected to a multistep minimization and equilibration protocol prior to production molecular dynamics (MD) simulations. First, energy minimization was performed for 1000 cycles using the steepest descent algorithm with a harmonic positional restraint of 10 kcal/mol·Å^2^ applied to all protein atoms. The system was then gradually heated over 1 ns using a 1 fs timestep: 500 ps from 100 K to 310 K, followed by 500 ps at 310 K under constant volume conditions. This was followed by 500 ps of equilibration at 310 K using a Monte Carlo [20] barostat with positional restraints of 10 kcal/mol·Å^2^ applied to all protein atoms. Subsequent equilibration was carried out through a staged reduction in positional restraints using a 1 fs timestep. Specifically, 500 ps was performed with restraints of 10 kcal/mol·Å^2^ on backbone heavy atoms and 5 kcal/mol·Å^2^ on side-chain atoms, followed by 500 ps with restraints reduced to 10 and 2.5 kcal/mol·Å^2^, respectively, and an additional 500 ps with restraints of 10 and 1.0 kcal/mol·Å^2^. A final equilibration step of 500 ps was performed with restraints of 10 kcal/mol·Å^2^ applied only to backbone heavy atoms. Production MD simulations were then conducted for 100 ns using a 2 fs timestep at 310 K under constant pressure with a Monte Carlo barostat. The SHAKE [21,22] algorithm was applied to constrain all bonds involving hydrogen atoms. A nonbonded cutoff of 8 Å was used, and long-range electrostatics were treated using the particle mesh Ewald (PME) [23] method. Temperature control was maintained using a Langevin thermostat [24] with a collision frequency of 2.0 ps^−1^. Backbone heavy atoms were restrained with a force constant of 10 kcal/mol·Å^2^ during production simulations.

### 2.3. Trajectory Analysis

Trajectory processing and analysis were performed using the cpptraj [25] module of AMBER. Protein stability was assessed by calculating the root-mean-square deviation (RMSD) of protein backbone heavy atoms (Figure S1). Binding free energies between protein subunits were estimated using the Molecular Mechanics/Poisson–Boltzmann Surface Area (MM/PBSA) [26] method. For these calculations, the IL-10 ligand and IL-10 receptor α subunit were defined as the receptor, while the IL-10 receptor β subunit was defined as the ligand. MM/PBSA analysis was performed on the final portion of each trajectory, corresponding to frames 250–1250 of a 25 ns simulation. Every fifth frame was extracted, yielding 200 snapshots per system. Calculations were conducted using a salt concentration of 150 mM and an internal dielectric constant of 4.0. Entropic contributions were not explicitly evaluated.

### 2.4. Animals

The mice used in this study were purchased from Taconic Biosciences (Rensselaer, NY, USA). IL-10 knockout (IL-10 KO) mice developed on a Balb/c background (BALB/cAnNTac-Il10em7Tac) spontaneously develop a high incidence of IBD by 24 weeks of age. Male IL-10 KO and Balb/c mice (as a normal control) were received at 8 weeks of age. The animals were evaluated for IBD Activity Index (DAI) scoring as a baseline. The DAI score was calculated based on body weight loss, stool consistency, and the presence of blood in the stool or around the rectum [27]. Additionally, blood samples were collected as a pretreatment control. The IL-10 KO mice were IP-injected with saline (saline group), or rAAV8 virus vectors expressing GFL (GFP group as a vector control), wild-type IL-10 (W group), or IL-10A (A group), IL-10B (B group) or IL-10C (C group). Animals were randomly assigned into treatment groups. The number of animals per group was determined based on previous studies. Starting from 10 weeks of age (or 2 weeks after the vector injection), IBD development (DAI score) assessment was performed twice a week, and blood samples were collected every other week until the end of the experiment (24 weeks of age). The animal study protocol (202111583) was approved on 20 February 2022 by the institutional animal care and use committee (IACUC) of the University of Florida (UF-IACUC).

### 2.5. Plasmid Constructs and Recombinant Adeno-Associated Virus Vectors (rAAV8)

With computational modeling of the interaction between human IL-10 and IL-10R β-subunit, we identified two candidate reaction sites and made mutations at these sites, designated as IL-10 mutant A and IL-10 mutant B. We also generated IL-10 mutant C (mutation on both sites). The coding sequences for wild-type human IL-10 (W) and the mutants (A, B, and C) were codon-optimized and synthesized including cloning sites (HindIII for 5′ and NotI for 3′). We also inserted one unique restriction enzyme site for each gene for identification purposes (Sph1 for A, BglII for B, and XhoI for C). These synthetic genes were inserted into an AAV-ITR containing plasmid (pTR-CB-AAT) by replacing the AAT gene from HindIII (5′) to NotI (3′) [28]. In each plasmid, the W, A, B, or C gene is driven by the CMV enhancer chicken-beta actin promoter (CBp). For in vitro function testing, these plasmids were transfected into 293 cells using Lipofectamine 3000 (ThmoFisher Scientific, Waltham, MA, USA). The transfected cells were cultured in serum-free medium for 2 days. The culture medium was collected to detect the concentration of W, A, B, or C and was used for in vitro experiments. For in vivo experiments, rAAV8 vectors were packaged at PackGene (Houston, TX, USA) using the corresponding plasmids.

### 2.6. Cell Culture and Detection of Luciferase Activity

In this study, we used three cell lines: THP-1 (human monocytes, TIB-202™, ATCC), U-937 (human monocytes, CRL-1593.2™, ATCC), and RAW 264.7 (mouse macrophages, TIB-71™, ATCC). THP-1 cells were cultured in RPMI 1640 with 10% FBS, 1% GlutaMAX, 1% penicillin-streptomycin, 0.05 mM of 2-mercapoethanol, and 1% MEM Nonessential Amino Acids (100×). U-937 cells were cultured in RPMI 1640 with 10% FBS, 0.05 mM of 2-mercapoethanol, and 1% penicillin-streptomycin. RAW 264.7 cells were cultured in DMEM with 10% FBS, 4.6 g/L glucose L-glutamine and sodium pyruvate, 1% penicillin-streptomycin, 1% MEM Nonessential Amino Acids, and 1% GlutaMAX. All cells were cultured in an incubator at 37 °C and 5% CO_2,_ and standard cell culture protocols were utilized for adherent and suspension cells.

A Lentivirus vector carrying a pSTAT3 responsive luciferase reporter gene was purchased from BPS Bioscience (6405 Mira Mesa Blvd Suite 100 San Diego, CA 92121, USA). This vector was used to infect (at MOI = 10) THP-1, U-937, and RAW 264.7 cells to generate THP-Luc, U-Luc, and Raw-Luc cells. The transduced cells were selected by culturing them in puromycin containing (0.5 μg/mL) medium for 3 weeks.

For detecting luciferase activity, THP-Luc, U-Luc, and Raw-Luc cells were seeded in 96-well plates at 60,000 cells per well and were treated with W, A, B, or C containing medium for 5 h, and then D-luciferin was added for 15 min and was read via luminescence reading on a microplate reader at 578 nm. A blank reading of the plate was done before the addition of D-luciferin and was used to eliminate the background reading of the plate.

### 2.7. ELISAs

For the detection of W, A, B, and C in culture medium or in serum, an IL-10 ELISA kit (Invitrogen, Carlsbad, CA, USA) was used following the manufacturer’s instructions. For the detection of lipocalin-2 in stool samples, a Lipocailin-2 ELISA kit (R & D Systems, Minneapolis, MN, USA) was used following the manufacturer’s instructions. Briefly, the stool sample from each mouse was weighed and placed in a tube with 1 mL of PBS. Tubes were placed on a vortex machine for 30 min at high speed to break up the stool. After the vortex, tubes were placed at 4 °C overnight. Tubes were centrifuged at 10,000× *g* for 10 min and then the supernatant was transferred into a new 1.5 mL microcentrifuge tube and stored in a −80 °C freezer until use. For the detection of anti-human IL-10 antibodies, we developed an ELISA assay. Briefly, IL-10 (expressed in 293 cells) in 100 μL of Voller’s buffer was coated on a 96-well ELISA plate at 4 °C overnight. The plate was washed three times with 200 μL of PBS-Tween 20 and blocked with 100 μL BSA at 37 °C for 1 h. Diluted mouse serum samples were then incubated at 37 °C for 1 h. Mouse IgG was used as a standard. After washing, 5000-time diluted goat-ant-mouse IgG-HRP (Invitrogen, Carlsbad, CA, USA) was incubated at 37 °C for 1 h. After the incubation and washing, OPD substrate (Sigma Aldrich, Saint Louis, MO, USA) solution (100 μL) was used and the reaction was terminated by sulfuric acid. The plate was read at 490 nm.

### 2.8. Flow Cytometry

A Cytek Aurora 5L was utilized to conduct the flow cytometry. The associated antibodies used were PE (Invitrogen, Carlsbad, CA, USA) for pSTAT3, Alexa Flour 700 (Invitrogen, Carlsbad, CA, USA) for CD3, LIVE DEAD NIR (Invitrogen, Carlsbad, CA, USA) for viability, BV711 (Bio Legend, San Diego, CA, USA) for CD4, PercP (BD Horizon, San Jose, CA, USA) for CD8a, BV 605 (BioLegend, San Diego, CA, USA) for CD335, CD122 for BUV 661 (BD Horizon, San Jose, CA, USA), Ly6c for FITC (Bio Legend, San Diego, CA, USA), CD11b for PercP-Cy 5.5 (Bio Legend, San Diego, CA, USA), and TCRb for RB744 (BD OptiBuild, San Jose, CA, USA).

### 2.9. Histological Processing and Evaluation

After sacrificing, the colon from each mouse was harvested (from the cecum to the rectum). The colon length and weight were measured. The colon was rinsed with saline to remove stools in the colon. We weighed this colon again to get a clean weight for each colon sample. The clean colon was processed into a Swiss-roll and fixed in 10% formalin. The fixed Swiss-roll was paraffin-embedded for sectioning. The colon sections were subjected to H&E staining.

The H&E-stained colons were evaluated using a common histological scoring system [29,30]. The scoring system semi-quantitatively evaluates infiltration, goblet cell loss, crypt density, crypt hyperplasia, muscle thickening, submucosal infiltrate, crypt abscess, and ulceration. Each criterion was scored from 0 to 3, except for crypt abscess and ulceration, which was scored as either 0 or 3. The histological score for each animal was calculated by adding all the scores in each criterion and was indicative of the severity of colon damage due to IBD. All evaluations were performed in a blinded fashion with respect to treatment group.

### 2.10. Statistics

Data analysis was performed in GraphPad Prism 10.0 software. One-way ANOVA, with correction for multiple comparisons, was used to compare differences among groups. Results are expressed as means ± standard deviation for all the analyses conducted in this paper. Statistical significance was indicated as follows: * *p* < 0.05, ** *p* < 0.01, *** *p* < 0.001, or the absence of a common superscript letter between two groups indicates a significant difference (*p* < 0.05).

## 3. Results

### 3.1. Computational Design of IL-10 Mutants

**Figure 1:**
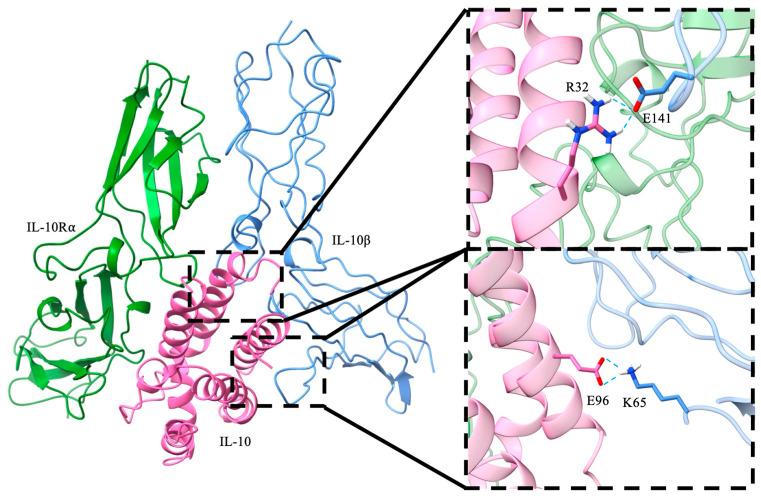
Overall structure of the IL-10 complex bound to the IL-10 receptor α and β subunits. Enlarged views highlight residues R32 and E96, which were selected for mutational analysis.

**Figure 2:**
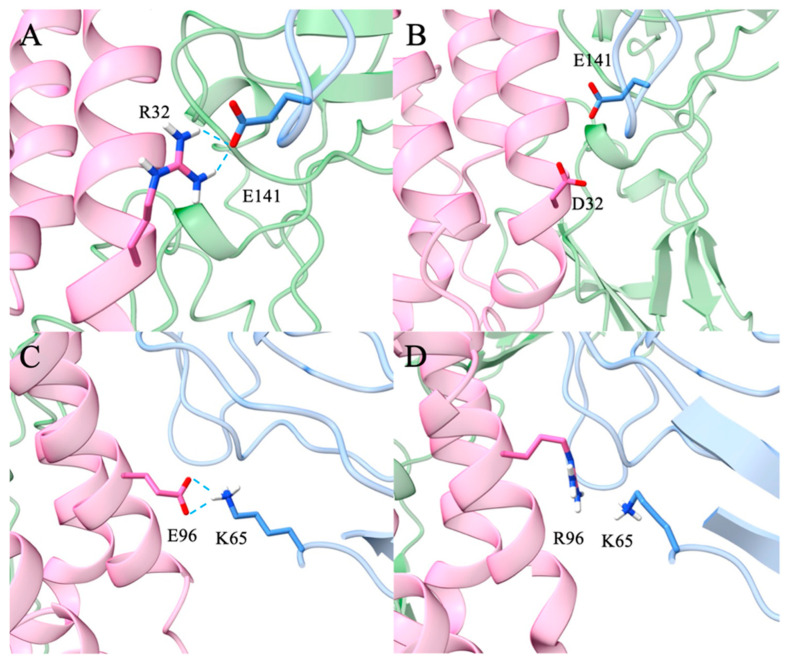
Interactions between human IL-10 mutants and human IL-10 Rβ. (**A**) Electrostatic and hydrogen-bond interactions between wild-type IL-10 residue R32 and IL-10Rβ residue E141; (**B**) Corresponding interaction in the R32D mutant; (**C**) Electrostatic and hydrogen-bond interactions between wild-type IL-10 residue E96 and IL-10Rβ residue K65; (**D**) Corresponding interaction in the E96R mutant.

**Figure 3:**
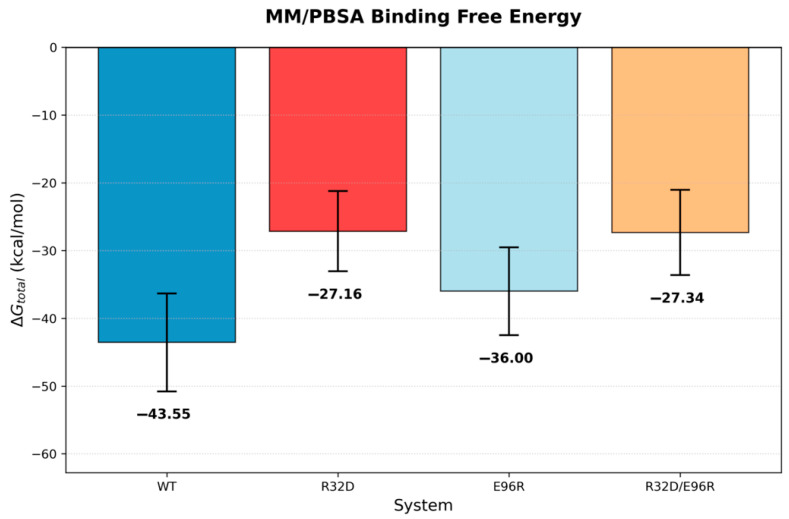
Average MM/PBSA binding free energies (kcal/mol) ± standard deviation computed from the final 20 ns of the MD trajectories for human IL-10 ligand and human IL-10 receptor WT, R32D, E96R, and R32D/E96R systems.

**Figure 4:**
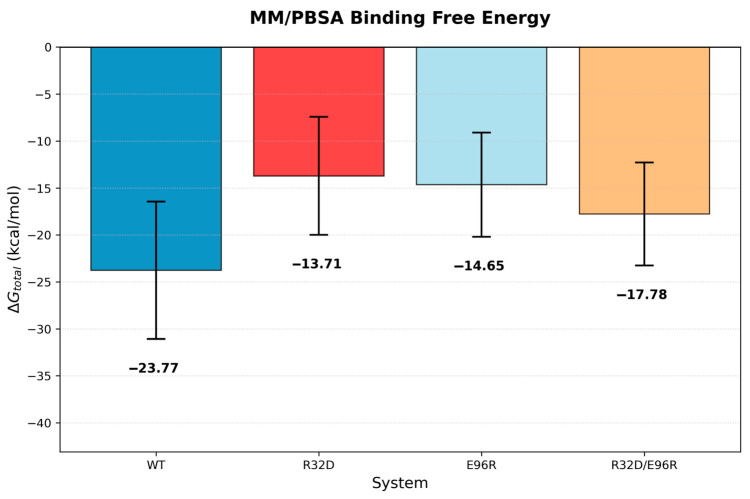
Average MM/PBSA binding free energies (kcal/mol) ± standard deviation computed from the final 20 ns of the MD trajectories for human IL-10 ligand and mouse IL-10 receptor WT, R32D, E96R, and R32D/E96R systems.

IL-10 has both anti-inflammatory and pro-inflammatory functions, which makes it challenging to use IL-10 as a therapeutic drug. Based on structural information of the IL10/IL-10Rα/IL10Rβ, our strategy was to mutate IL-10 residues that interact with the D1 and D2 domains of IL-10Rβ to reduce the pro-inflammatory effect. We identified the following IL-10 mutants that are projected to have better therapeutic potential: D25R, E96R, R32D, R32E, R32D-E96R, R32D-E96K, R32E-R96RE96R, R32E-E96K, D25K-E96K, D25K-E96R, and D25R-E96K. Here we describe and test three of them (R32D, E96R, and R32D-E96R). In the wild-type complex, IL-10 residue R32 forms a charge–charge interaction with E141 of the IL-10 receptor β subunit, whereas E96 engages in an electrostatic interaction with K65 of the β subunit (Figure 1). These residues were targeted for mutation to selectively weaken local electrostatic contacts with the β subunit without fully abolishing receptor binding, thereby enabling evaluation of the contribution of these interactions to complex stability and binding energetics. The mutations examined are located at the local IL-10–IL-10Rβ binding interface, which is structurally self-contained within each trimer and does not require the presence of the full hexamer to be accurately represented. Upon mutation at both sites, repulsive electrostatic interactions were observed between the mutated residues and their corresponding interaction partners, resulting in reduced interfacial contacts (Figure 2). To quantify the contribution of these residues to overall complex stability, binding free energies between the subunits were estimated using MM/PBSA. Explicit entropy calculations are computationally intensive and were therefore not included in the MM/PBSA analysis, which was restricted to enthalpic contributions. For closely related systems such as those studied here, entropic contributions are expected to be similar in magnitude; thus, relative binding trends can still be meaningfully interpreted despite the absence of explicit entropy calculations. The wild-type system exhibited a binding free energy of −43.56 ± 7.23 kcal/mol. Relative to the wild-type, the R32D mutant showed a substantial reduction in binding affinity to −27.16 ± 5.92 kcal/mol, while the E96R mutant displayed a more moderate decrease to −36.00 ± 6.49 kcal/mol (Figure 3). The R32D/E96R double mutant yielded a binding free energy of −27.34 ± 6.30 kcal/mol, comparable to the R32D single mutant. Collectively, these results indicate that mutation of R32 exerts a more pronounced impact on β-subunit binding than mutation of E96, consistent with the dominant stabilizing contribution of the R32–E141 interaction at the interface. Notably, the in silico binding studies using the human ligand and mouse receptor yielded results consistent with those observed in the other systems (Figure 4 and Figure S2).

### 3.2. Functional Evaluations In Vitro Using Human and Mouse Cells

**Figure 5:**
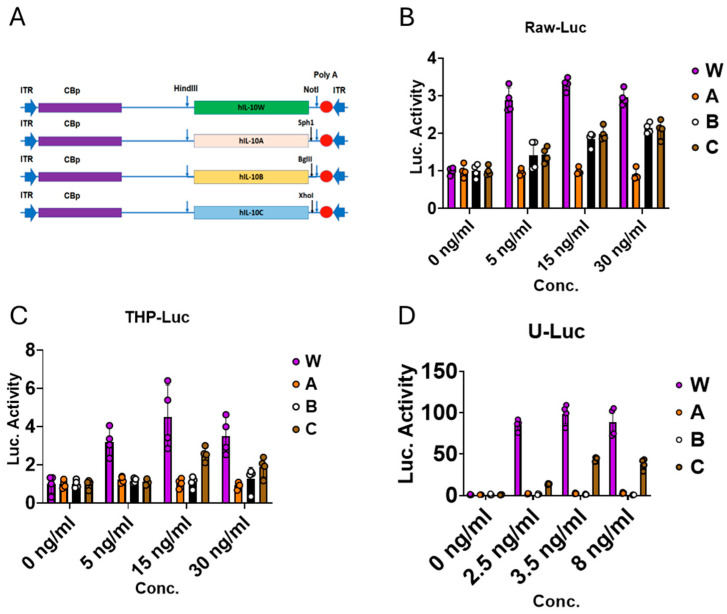
Constructs for expressing IL-10 wild-type (W) and mutants (A, B, and C) and functional tests in human and mouse cells. (**A**) Plasmid constructs for expressing IL-10 wild-type (W) and mutants (A, B, and C); (**B**–**D**) Reporter gene (luciferase) activities responding to the treatment of IL-10 and its mutants (W, A, B, and C) expressed in HEK 293 cells. RAW-Luc (C, mouse macrophages), THP-Luc (D, human monocytes), and U-Luc (E, human monocytes). *n* = 4 for each group of W, A, B, and C.

To evaluate the selected IL-10 mutants, we synthesized the corresponding coding genes and cloned them into expression plasmids. These constructs encoded wild-type human IL-10 (designated W) or the mutants: A (R32D), B (R96), and C (R32D/E96R), as depicted in Figure 5A. We overexpressed W, A, B and C in HEK293 cells by plasmid transfection. Protein concentrations were quantified by an IL-10 ELISA.

We next generated three reporter cell lines (Raw-Luc, THP-Luc, and U-Luc) from RAW 264.7, THP-1, and U937 cells by transducing them with a lentiviral vector that encodes luciferase driven by a pSTAT3-responsive promoter. The reporter cells were treated with a range of concentrations of protein W, A, B, or C, each produced in 293 cells. Figure 5B–D show that the IL-10 mutants elicit lower reporter activity than wild-type IL-10. Remarkably, human IL-10 is functional in mouse cells, as illustrated by the Raw-Luc line (a mouse monocyte line) in Figure 5B. These results provide strong support for testing these human proteins in mouse models.

### 3.3. IL-10 Gene Therapy Reduced IBD Development in IL-10 KO Mice

**Figure 6:**
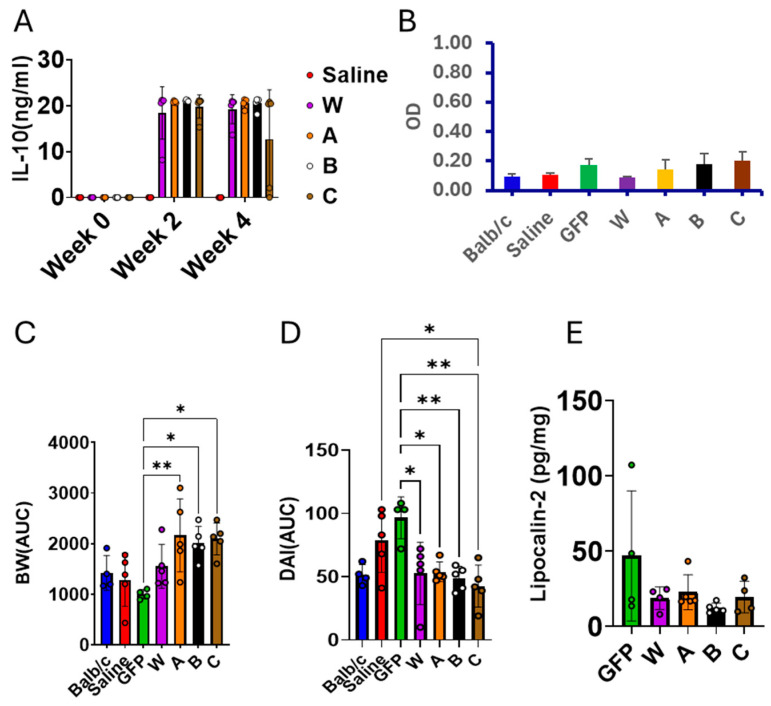
Effect of IL-10 (W, A, B, and C) gene therapy on IBD development in IL-10 KO mice. (**A**) Serum levels of W, A, B, and C detected by IL-10 ELISA; (**B**) Anti-hIL-10 levels detected by ELISA at 12 weeks post vector injection; (**C**) Body weight changes in all groups. Area under the curve (AUC) was calculated for each animal using data in all time points; (**D**) the effect of IL-10 gene therapy on IBD development; (**E**) Lipocolin-2 levels in the stool samples detected by ELISA. Balb/c mice (*n* = 4) as a normal mice control. Saline, (*n* = 5) IL-10 KO mice injected with saline as a disease model control. GFP, (*n* = 4) IL-10 KO mice injected with rAAV8-GFP as a vector control. W (*n* = 4), A (*n* = 5), B (*n* = 5), and C (*n* = 5), IL-10 KO mice injected with rAAV vectors expressing W, A, B, and C, respectively. AUC, area under curve. * *p* < 0.05, ** *p* < 0.01.

To evaluate the therapeutic potential of our IL-10 mutants, we administered gene therapy to IL-10 knockout (KO) mice that spontaneously develop inflammatory bowel disease. IL-10 KO cohorts (8 weeks of age) were intraperitoneally injected with either saline (model control) or rAAV8 vectors (10^11^ vg/mouse) expressing wild-type IL-10 (W), mutant A (R32D), mutant B (R96), mutant C (R32D/E96R), or GFP (vector control). Saline-injected BALB/c mice served as normal controls. As shown in Figure 6A, all vectors produced high, sustained transgene expression at 2 and 4 weeks post-injection. Human IL-10 proteins delivered by rAAV8 elicited minimal or negligible immune responses (Figure 6B). Body weight trajectories and the disease activity index (DAI) were monitored twice weekly up to 24 weeks. Compared with GFP controls, the A, B, and C groups displayed significantly greater weight gain (area under the curve, AUC) (Figure 6C and Figure S3). The DAI AUC was significantly lower in the W, A, B, and C groups than in the GFP group, and roughly matched that of the normal BALB/c cohort (Figure 6D and Figure S4). Fecal lipocalin-2 concentrations were reduced in the W, A, B, and C groups relative to GFP (Figure 6E). Together, these results clearly indicate that the IL-10 (W, A, B, and C) gene therapy can correct the IL-10-deficiency-associated disease development.

### 3.4. IL-10 Gene Therapy Reduced Colon Injury in IL-10 KO Mice

**Figure 7:**
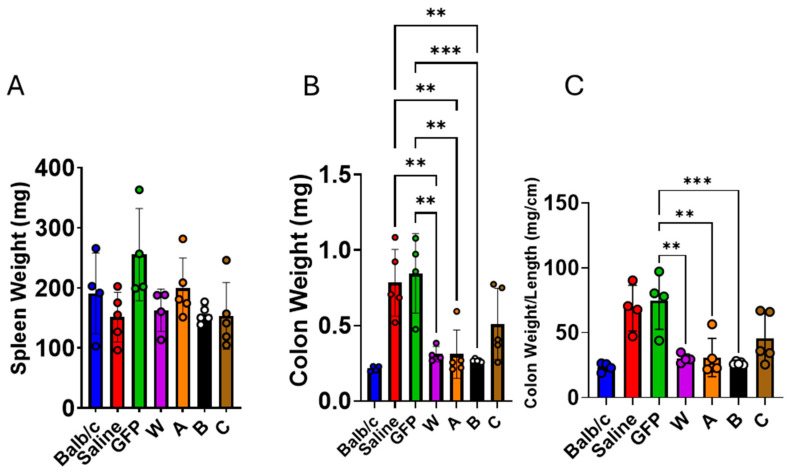
Effect of IL-10 (W, A, B, and C) gene therapy on spleen and colon weight in IL-10 KO mice. (**A**) Spleen weight; (**B**) Clean (PBS rinses) colon weight; (**C**) ratio of colon weight and length. Balb/c mice (*n* = 4) Saline, (*n* = 5) IL-10 KO mice injected with saline as a disease model control. GFP, (*n* = 4) IL-10 KO mice injected with rAAV8-GFP as a vector control. W (*n* = 4), A (*n* = 5), B (*n* = 5), and C (*n* = 5), IL-10 KO mice injected with rAAV vectors expressing W, A, B, and C, respectively. ** *p* < 0.01, *** *p* < 0.001.

**Figure 8:**
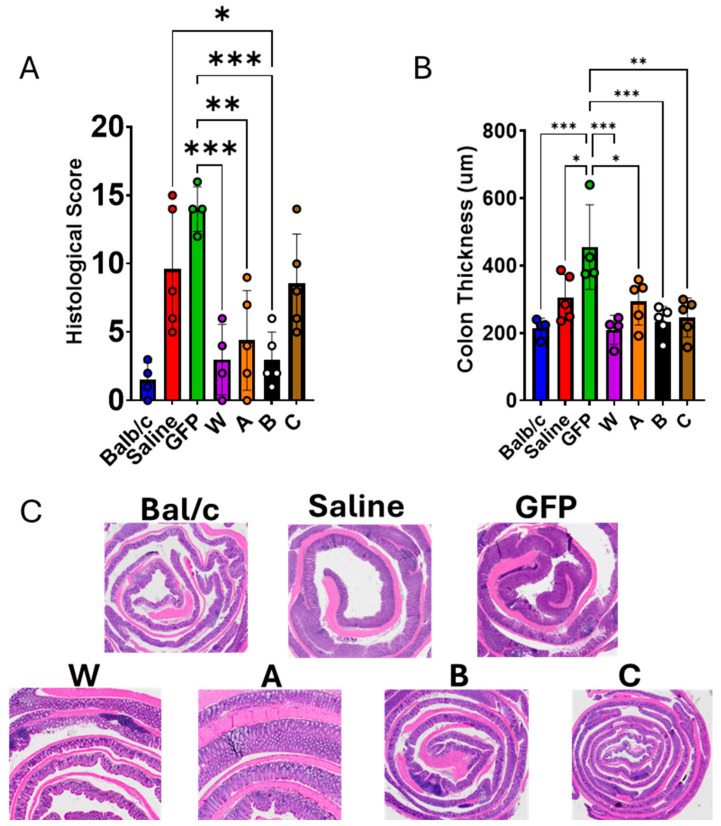
Effect of IL-10 (W, A, B, and C) gene therapy on colon injury in IL-10 KO mice. (**A**) The average colon histological scores in each group; (**B**) The average colon thickness in each group; (**C**) The representative images of H&E-stained colon sections from each group. Balb/c mice (*n* = 4) as a normal mice control. Saline, (*n* = 5) IL-10 KO mice injected with saline as a disease model control. GFP, (*n* = 4) IL-10 KO mice injected with rAAV8-GFP as a vector control. W (*n* = 4), A (*n* = 5), B (*n* = 5), and C (*n* = 5), IL-10 KO mice injected with rAAV vectors expressing W, A, B, and C, respectively. * *p* < 0.05, ** *p* < 0.01, *** *p* < 0.001.

At 24 weeks of age (16 weeks after vector injection), all mice were sacrificed for histological and immunological analysis. The mean spleen weight in the GFP group was slightly higher than in the other groups, but the difference was not statistically significant—likely reflecting the modest sample size (Figure 7A). In contrast, the average colon weight in the W, A, and B groups was significantly lower than in the GFP and saline controls (Figure 7B,C). Hematoxylin–eosin (H&E)-stained colon sections were scored for inflammation. As shown in Figure 8A, the control groups displayed markedly higher histological scores than the W, A, and B groups. Additionally, colon thickness was significantly greater in the GFP group compared with the W, A, B, and C groups (Figure 8B,C). Collectively, these findings demonstrate that IL-10 gene therapy markedly protects against colon injury in IL-10 KO mice.

### 3.5. IL-10 Mutants Have Lower Activities in Stimulating NK and CD8+ T Cells

**Figure 9:**
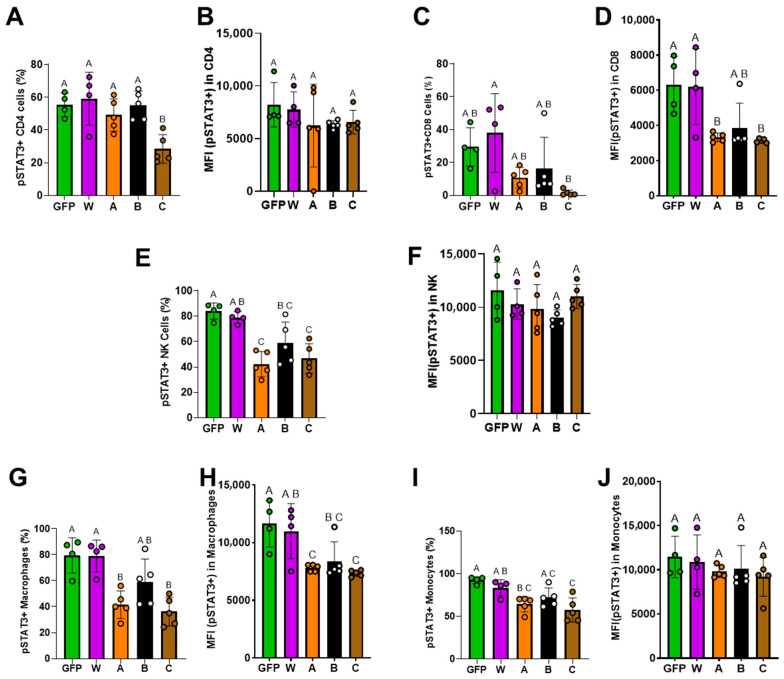
Effect of IL-10 (W, A, B, and C) gene therapy on pSTAT3 levels in immune cells in IL-10 KO mice. Percentage of pSTAT3+ cells and pSTAT3 intensity (MFI) in each cell population were detected by flow cytometry. (**A**,**B**) in CD4 T cells; (**C**,**D**) in CD8 T cells; (**E**,**F**) in NK cells; (**G**,**H**) in macrophages; (**I**,**J**) in monocytes. GFP, (*n* = 4) IL-10 KO mice injected with rAAV8-GFP as a vector control. W (*n* = 4), A (*n* = 5), B (*n* = 5), and C (*n* = 5), IL-10 KO mice injected with rAAV vectors expressing W, A, B, and C, respectively. Data without a common superscript letter are different (*p* < 0.05) among treatment groups.

We isolated splenocytes from each animal and performed flow cytometry analysis to detect pSTAT3 levels as an indicator of IL-10 signaling (Figure S5). As shown in Figure 9, IL-10 mutants and wtIL-10 (W, A, B, and C) have similar effects on pSTAT3 levels in the CD4+ T cells (Figure 9A,B) and monocytes (Figure 9I,J). However, the mean fluorescence intensity (MFI) of pSTAT3 in CD8^+^ T cells was significantly lower in the A and C groups than in the W group (Figure 9C,D). Similarly, the frequncy of pSTAT3^+^ NK cells in the A and C groups was significantly lower than in the W group (Figure 9E), although pSTAT3 intensities (MFI) on NK cells were similar among these groups (Figure 9F). Notably, both the frequency and MFI of pSTAT3 in macrophages were markedly decreased in the A and C groups compared with W (Figure 9G,H). Together, these results demonstrated that the IL-10 mutants altered IL-10 signaling intensity in these cells. As CD8 T cells, NK cells, and macrophages play important roles in promoting the pro-inflammatory effect [31,32,33], these results suggest that our IL-10 mutants may potentially have a lower pro-inflammatory effect than wtIL-10.

**Figure 10:**
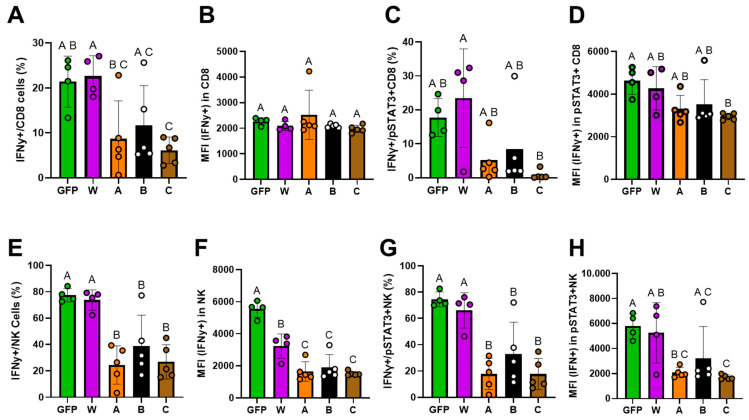
The effect of IL-10 mutants on IFN-γ expression in CD8 T and NK cells. The percentage of IFN-γ+ cells and FN-γ intensity (MFI) were detected by flow cytometry in the CD8+ T cells (**A**–**D**) and NK cells (**E**–**H**) (**A**,**B**) in the CD8 T cell population. (**C**,**D**) in pSTAT3+ CD8 T cells. (**E**,**F**) in the NK cells. (**G**,**H**) in the pSTAT3+ NK cells. Data without a common superscript letter are different (*p* < 0.05) among treatment groups.

While STAT3 phosphorylation is a canonical downstream event of IL-10 receptor signaling, IFN-γ is one of the key effector molecules for pro-inflammatory activity in CD8^+^ T and NK cells. We therefore examined whether IL-10 gene therapy modulates IFN-γ expression in these cell types. As shown in Figure 10A,C, the frequency of IFN-γ+ CD8 and IFN-γ+ pSTAT3+ CD8 cells was significantly lower in the IL-10 mutant (A and C) groups than in the W group, whereas the IFN-γ levels (MFI) on these cells were not affected by the treatments (Figure 10B,D). Remarkably, IFN-γ expression (both frequency and intensity) in NK cells and pSTAT3 + NK cells was singificantly lower in all IL-10 mutant (A, B, and C) groups than in the W group. These data demonstrate that our IL-10 mutants possess a substantially lower capacity to stimulate effector T and NK cell IFN-γ production compared with wild-type IL-10.

## 4. Discussion

IL-10 is a bifunctional, or double-edged cytokine. It can act on myeloid cells (monocytes and macrophages) and mediate anti-inflammatory effects [34,35]. It can also act on effector cells (CD8+ T cellss, and NK cells) and mediate pro-inflammatory effects through stimulating IFN-γ and granzyme B expressions [7,8]. These bifunctional effects make it challenging to use IL-10 as an anti-inflammatory drug. Interestingly, two factors play an important role in altering these opposing biological effects of IL-10: (1) the density of the receptor subunits on the target cells; and (2) the affinity of IL-10 to the receptor subunits. It has been shown that the IL-10Rβ expression in CD8+ cells is much lower than that in macrophages [9]. In addition, IL-10 has a lower affinity to IL-10Rβ than to IL-10Rα [36]. These differences provide a reason why IL-10 may have a stronger anti-inflammatory effect and a relatively lower pro-inflammatory effect. These differences also provide an opportunity to create IL-10 mutants for eliminating the pro-inflammatory effect by weakening the interactions between IL-10 and IL-10 Rβ. In this study, we used computational design technology to identify two IL-10 residues that are critical for interacting with IL-10Rβ and created three IL-10 mutants. Results from this study showed that these IL-10 mutants have lower activity in stimulating CD8+ T cells and NK cells compared with wtIL-10, while having a similar function in correcting IL-10 deficiency. Although these findings in the IL-10 KO model do not conclusively demonstrate a reduced pro-inflammatory effect, they suggest that these IL-10 mutants may have therapeutic potential for inflammatory diseases including IBD. These results also provide insight for altering the functions of other protein drugs.

Phosphorylated STAT3 (pSTAT3) is an important cellular protein mediating IL-10 signal transduction in both myeloid and effector cells, thus serving as a marker for the functional evaluation of IL-10 mutants. In this study, we generated pSTAT3-responsive reporter cell lines and showed lower activities when treated with IL-10 mutants than when treated with wtIL-10. Importantly, in IL-10 KO mice, IL-10 mutants corrected the IL-10 defect related to IBD development (DAI and colon injury) similar to IL-10W, but resulted in significantly lower pSTAT3 levels in T effector (CD8+) and NK cells. Furthermore, these effects extended to IFN-γ expression in CD8+ and NK cells. The mechanisms underlying these effects remain to be further investigated. It is possible that the mutants reduce pSTAT3 levels and, in turn, affect the viability of effector cells as described by Sun et al. [37] Although these results are important, the pro-inflammatory effect needs to be further investigated in other models, such as malignancy or specific infections, where exogenous IL-10 induces clinical worsening or the aberrant activation of effector cells like CD8+ T cells. In addition, pSTAT3 can mediate signal transduction for other cytokines (e.g., IL-6). In future studies, other markers may be used to evaluate the pro-inflammatory effects.

It has been reported that human IL-10 is functional on mouse cells [38], but mouse IL-10 is not functional on human cells [39]. Previous studies showed that mouse IL-10 gene therapy significantly prevented type 1 diabetes development in non-obese diabetes (NOD) mouse models by inducing CD4+CD25+ cells [40,41,42]. However, human IL-10 gene therapy in IL-10 KO mice has not been investigated. In this study, we showed that human IL-10 and its mutants are functional in mouse models, suggesting our IL-10-mutant-expressing rAAV vectors can be used in other mouse models such as DSS-induced colitis mouse models.

In this study, we used an rAAV8 vector to deliver human IL-10 mutants and observed a low immune response to the proteins. These results are consistent with our previous observations in several mouse disease models, including arthritis, lupus, and osteoporosis [43,44,45]. The possible underlying mechanisms include the fact that rAAV8 vectors fail to transduce dendritic cells (DCs), thereby inducing immune tolerance to the transgene products [10]. In addition, a single injection of an rAAV8 vector can mediate long-term and sustained transgene expression in liver or muscle cells [28]. This feature makes it a powerful tool for evaluating protein-drug function in animal models, especially compared with using recombinant proteins, which can be challenging to produce in large amounts. Our results in this study provide strong support for the strategy of using rAAV8 vectors in the development of protein drugs. It should be noted that rAAV8-mediated IL-10 delivery yields sustained levels of IL-10, which may differ in kinetics from the repeated injection of an IL-10 protein drug.

There are some limitations to this study: (1) We used the IL-10 KO mouse model and showed IL-10 mutants are functional in correcting IL-10-deficiency-induced IBD and exhibit reduced activity on inflammatory mediator cells (CD8+ T and NK cells). However, IL-10 KO mice primarily represent a model of spontaneous colitis due to a lack of IL-10 and may not be a suitable model to evaluate the pro-inflammatory properties of IL-10. To fully evaluate the anti-inflammatory effects of these mutants, inflammation models using wtIL-10 mice will be helpful to clearly observe the therapeutic potential against the endogenous baseline responses. In addition, as wtIL-10 is effective for correcting IL-10 deficiency, it may be difficult to show the advantages of the IL-10 mutants. Future studies using other disease models will be needed to evaluate the pro-inflammatory effect, toxicity, and dose–response of the IL-10 mutants. (2) The number of animals in each group was relatively small, and only male mice were used. Although the results clearly demonstrated some differences between wtIL-10 and the IL-10 mutants, larger cohorts including female mice in future studies would provide greater statistical confidence and more detailed results.

## 5. Conclusions

In conclusion, our results demonstrate the following: (1) computational protein design proved to be a powerful tool for engineering IL-10 variants with altered functional properties; (2) the engineered IL-10 mutants exhibited reduced activation of CD8^+^ T cells and NK cells relative to wild-type IL-10, suggesting a potential for lower pro-inflammatory activity while preserving anti-inflammatory efficacy. These mutants, therefore, represent promising candidates for developing safer, and more effective therapeutics for inflammatory diseases.; and (3) in the IL-10 KO mouse model, human IL-10 mutants delivered by rAAV8 fully corrected the disease phenotype, demonstrating that these mutants can effectively restore IL-10 function in vivo. Although additional studies are required to rigorously assess safety, efficacy, dose–response, and therapeutic potential in diverse disease models, our results indicate that both the IL-10 mutants and their rAAV-mediated delivery vectors hold significant promise for treating inflammation-associated disorders.

## 6. Patents

This work is covered by a patent (T19506/WGS, U1197.70253WO00, PCT/US2025/046026 IL-10 Mutants for Anti-Inflammatory Therapy).

## Data Availability

The original contributions presented in the study are included in the article, further inquiries can be directed to the corresponding authors.

## Supplementary Materials

The following supporting information can be downloaded at: https://www.mdpi.com/article/10.3390/biom16030482/s1. Figure S1. Time evolution of the root-mean-square deviation (RMSD) of protein backbone heavy atoms during production molecular dynamics simulations for (A) WT, (B) R32D mutant, (C) E96R mutant, and (D) R32D/E96R double mutant systems. Figure S2. Interactions between human IL-10 mutants and mouse IL-10 Rβ. (A) Electrostatic and hydrogen-bond interactions between wild-type IL-10 residue E96 and IL-10Rβ residue R68. (B) Corresponding interaction in the E96R mutant. (C) Electrostatic and hydrogen-bond interactions between wild-type IL-10 residue R32 and mouse IL-10 receptor. Note that R32 of human IL-10 does not interact with the beta subunit of the mouse IL-10 receptor. (D) Corresponding interaction in the R32D mutant. Figure S3. Body weight in all groups. (A) Average body weight in all groups. (B–E) Body-weight change (%) for the W, A, B, and C groups, using saline-injected and rAAV-GFP-injected groups as controls. Figure S4. Average DAI scores. (A) DAI of the W group. (B) DAI of the A group. (C) DAI of the B group. (D) DAI of the C group. DAI of GFP groups serve as controls in each panel. Figure S5: The gating strategy for flowcytometry analysis.
