# Supplementary material for: Functional Evaluation of Computationally Designed IL-10 in IL-10 KO Mice
Source: Biomolecules. 2026 Mar 23;16(3):482. doi: 10.3390/biom16030482 (PMC13023582; doi:10.3390/biom16030482)
Supplement: Supplementary file 1 [file biomolecules-16-00482-s001.zip › biomolecules-4155973-supplementary.pdf]

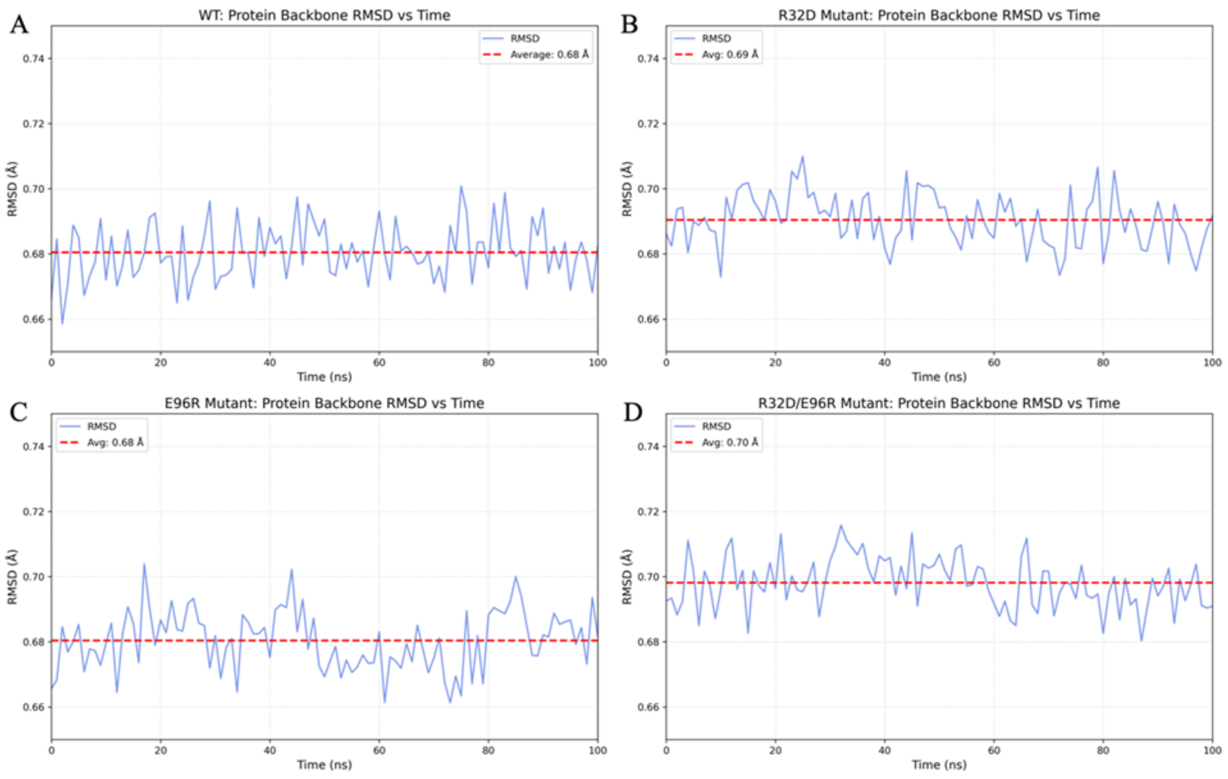

Figure S1. Time evolution of the root-mean-square deviation (RMSD) of protein backbone heavy atoms during production molecular dynamics simulations for (A) WT, (B) R32D mutant, (C) E96R mutant, and (D) R32D/E96R double mutant systems.

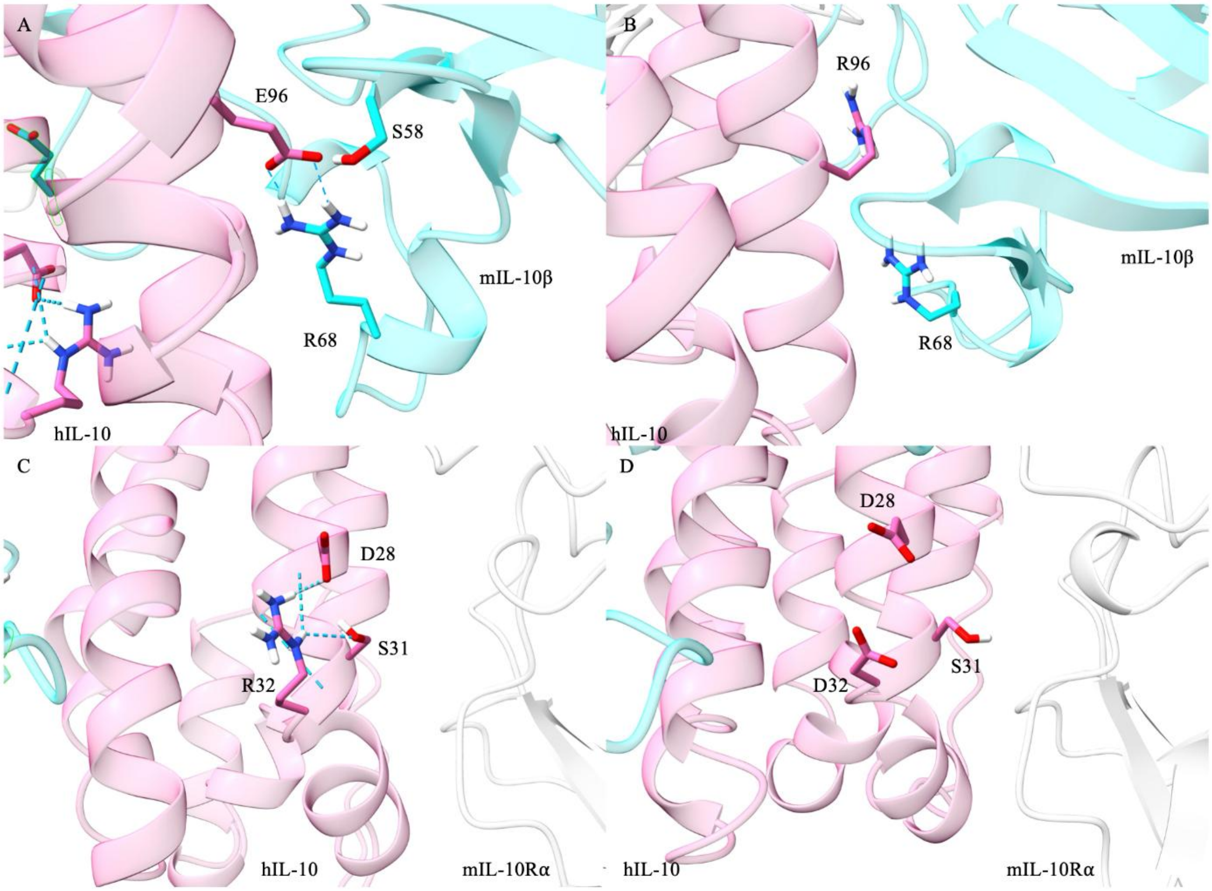

Figure S2. Interactions between human IL-10 mutants and mouse IL-10 Rβ. (A) Electrostatic and hydrogen-bond interactions between wild-type IL-10 residue E96 and IL-10Rβ residue R68. (B) Corresponding interaction in the E96R mutant. (C) Electrostatic and hydrogen-bond interactions between wild-type IL-10 residue R32 and mouse IL-10 receptor. Note that R32 of human IL-10 does not interact with the beta subunit of the mouse IL-10 receptor. (D) Corresponding interaction in the R32D mutant.

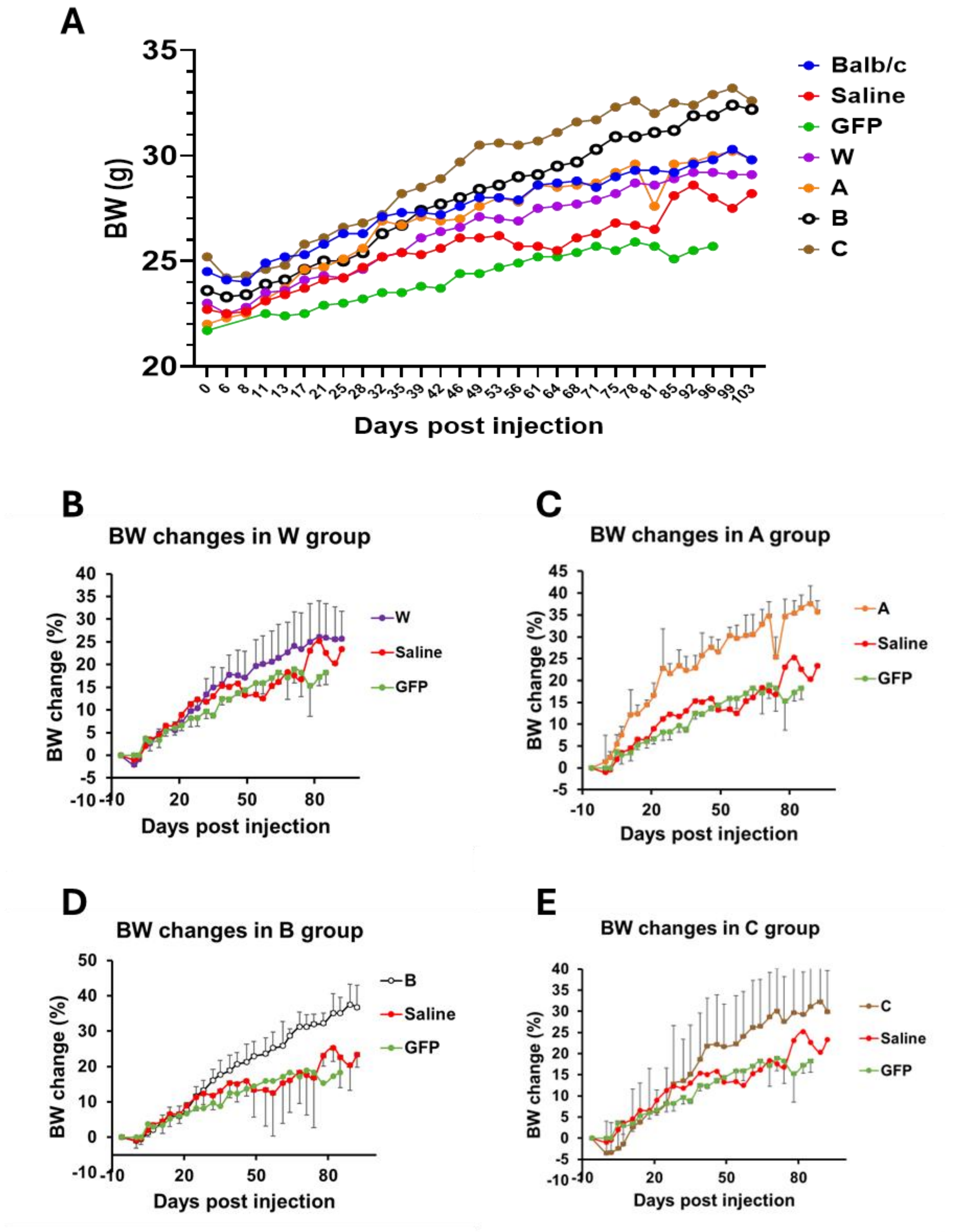

Figure S3. Body weight in all groups. (A) Average body weight in all groups. (B–E) Body-weight change (%) for the W, A, B, and C groups, using saline-injected and rAAV-GFP-injected groups as controls.

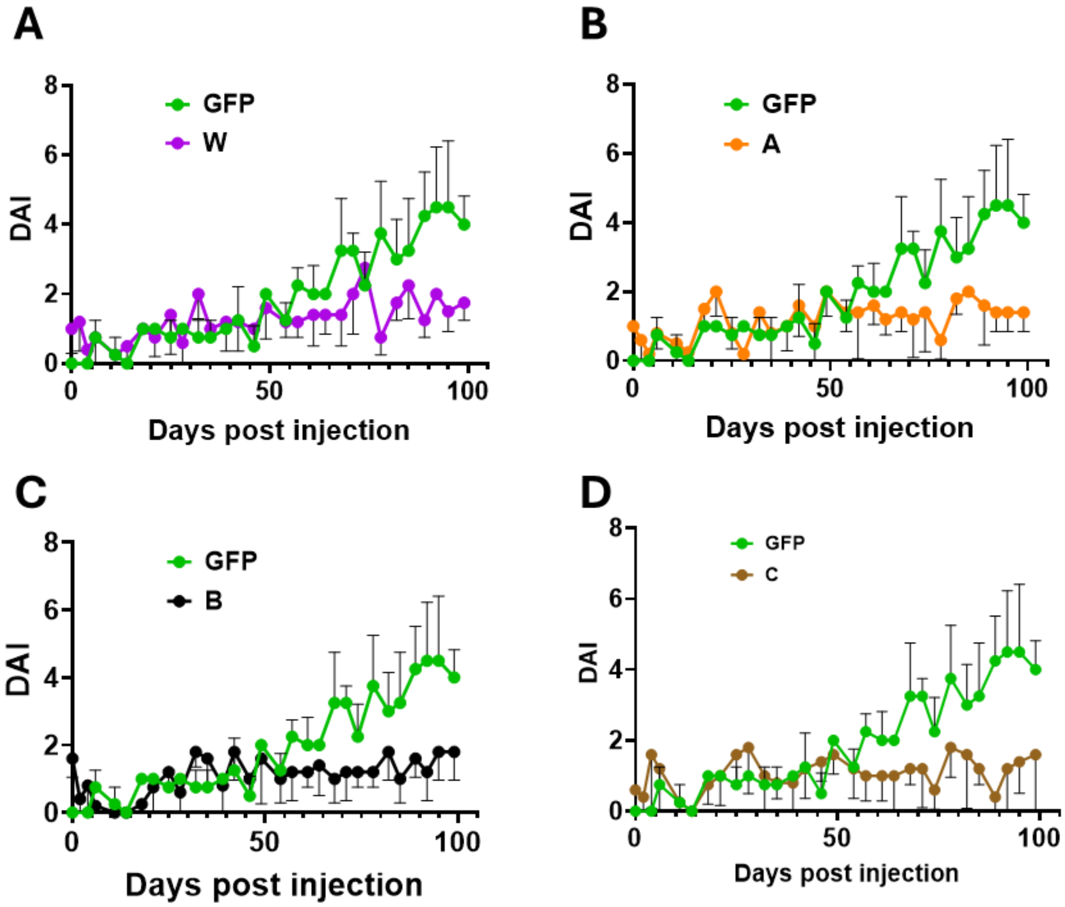

Figure S4. Average DAI scores. (A) DAI of the W group. (B) DAI of the A group. (C) DAI of the B group. (D) DAI of the C group. DAI of GFP groups serve as controls in each panel.

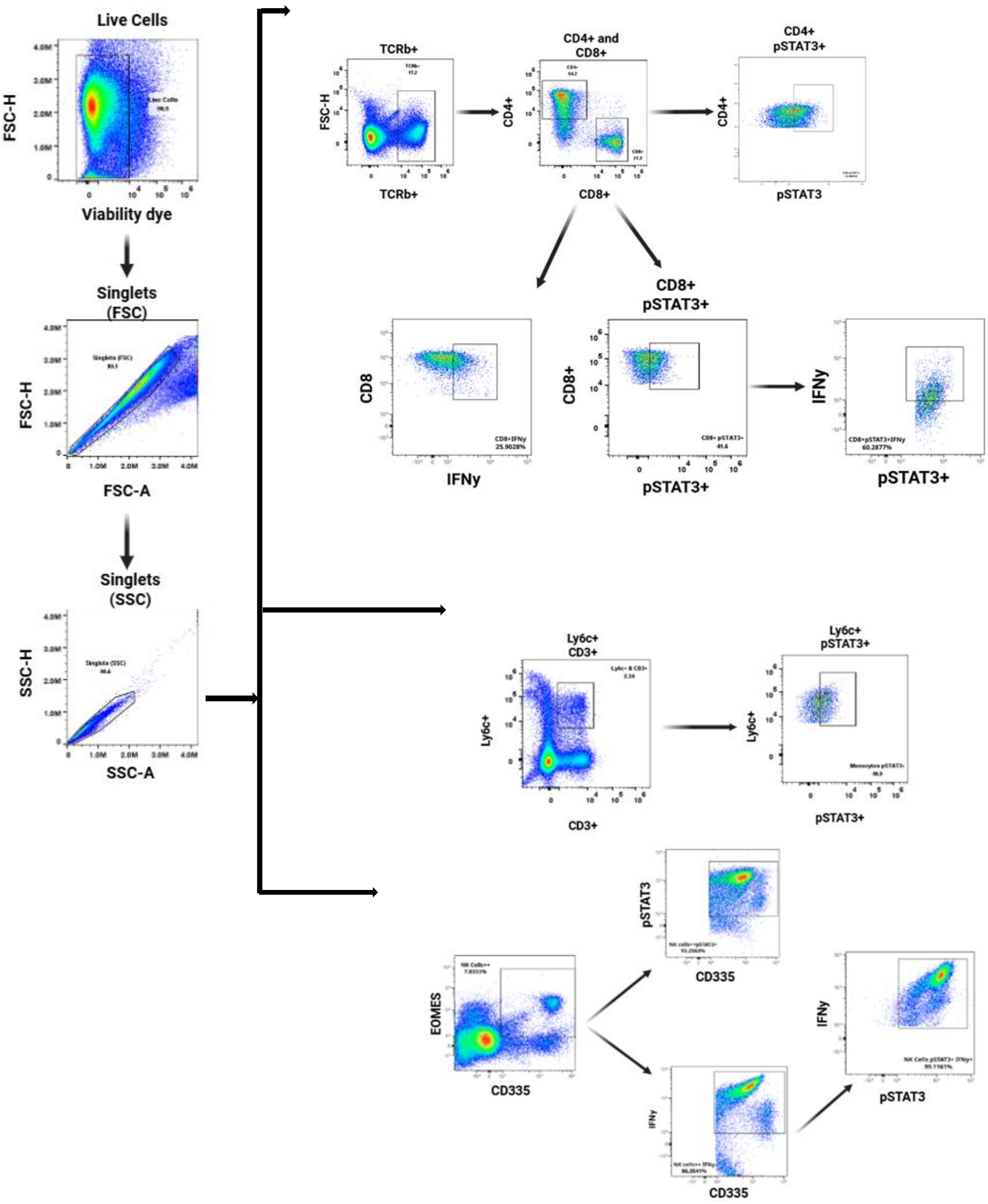

Figure S5: The gating strategy for flowcytometry analysis.
